# Consumption of sugar-sweetened beverages and T2D diabetes in the Eastern Caribbean

**DOI:** 10.1017/S1368980023000381

**Published:** 2023-07

**Authors:** Carol R Oladele, Neha Khandpur, Deron Galusha, Saria Hassan, Uriyoán Colón-Ramos, Mary Miller, Oswald P Adams, Rohan G Maharaj, Cruz M Nazario, Maxine Nunez, Rafael Pérez-Escamilla, Trevor Hassell, Marcella Nunez-Smith

**Affiliations:** 1 Equity Research and Innovation Center, Yale School of Medicine, 100 Church Street South, Suite A200, New Haven, CT 06510, USA; 2 Department of Nutrition, School of Public Health, University of São Paulo, Av. Dr. Arnaldo, São Paulo, Brazil; 3 Department of Nutrition, Harvard T.H. Chan School of Public Health, Boston, MA, USA; 4 Milken Institute School of Public Health, George Washington University, Washington, DC, USA; 5 The University of the West Indies, Cave Hill Campus, Barbados; 6 The University of the West Indies, St. Augustine Campus, Trinidad and Tobago; 7 Department of Biostatistics and Epidemiology, Graduate School of Public Health, University of Puerto Rico, Medical Sciences Campus, San Juan, Puerto Rico; 8 University of the Virgin Islands, School of Nursing, St. Thomas, VI, USA; 9 Yale University, School of Public Health, New Haven, CT, USA; 10 Healthy Caribbean Coalition, Bridgetown, Barbados

**Keywords:** Sugar-sweetened beverages, Sugar consumption, Type 2 diabetes, Caribbean

## Abstract

**Objective:**

Sugar-sweetened beverages (SSB) are implicated in the increasing risk of diabetes in the Caribbean. Few studies have examined associations between SSB consumption and diabetes in the Caribbean.

**Design:**

SSB was measured as teaspoon/d using questions from the National Cancer Institute Dietary Screener Questionnaire about intake of soda, juice and coffee/tea during the past month. Diabetes was measured using self-report, HbA1C and use of medication. Logistic regression was used to examine associations.

**Setting:**

Baseline data from the Eastern Caribbean Health Outcomes Research Network Cohort Study (ECS), collected in Barbados, Puerto Rico, Trinidad and Tobago and US Virgin Islands, were used for analysis.

**Participants:**

Participants (*n* 1701) enrolled in the ECS.

**Results:**

Thirty-six percentage of participants were unaware of their diabetes, 33% aware and 31% normoglycaemic. Total mean intake of added sugar from SSB was higher among persons 40–49 (9·4 tsp/d), men (9·2 tsp/d) and persons with low education (7·0 tsp/d). Participants who were unaware (7·4 tsp/d) or did not have diabetes (7·6 tsp/d) had higher mean SSB intake compared to those with known diabetes (5·6 tsp/d). In multivariate analysis, total added sugar from beverages was not significantly associated with diabetes status. Results by beverage type showed consumption of added sugar from soda was associated with greater odds of known (OR = 1·37, 95 % CI (1·03, 1·82)) and unknown diabetes (OR = 1·54, 95 % CI (1·12, 2·13)).

**Conclusions:**

Findings indicate the need for continued implementation and evaluation of policies and interventions to reduce SSB consumption in the Caribbean.

Caribbean populations have the highest burden from cardiometabolic risk factors including obesity and diabetes in the Americas and worldwide^(1)^. Prevalence estimates show that more than 60% of adults are overweight or obese, with prevalence exceeding 80% in some Caribbean countries^(2)^. Within the region, obesity prevalence is highest among women, who experience obesity at rates three times greater than men^(3)^. Diabetes, strongly associated with obesity, poses a serious burden to population health and healthcare systems in the region. With a range of rates between 9 and 15%^(3,4)^, the Caribbean has some of the highest rates of diabetes in the Americas, and the second highest rate among the seven regions of the International Diabetes Federation^(1)^.

The increasing secular trend in prevalence of obesity and diabetes is attributed to the nutrition transition underway in the region. The nutrition transition is likely related to a heavy reliance on globalised food production systems and imported food, as at least 80% of available food in several Caribbean countries is imported^(5)^. Estimates from the Food and Agricultural Organization of the United Nations show that food items that are ultra-processed – industrial formulations manufactured from substances derived from foods or other organic sources that typically contain added flavours, colours and other cosmetic additives^(6)^ – contribute significantly to food imports in the region^(5)^. The nutrition transition in this region is characterised by a shift in the dietary patterns to include more ultra-processed foods – typically high in added sugars, fats and Na. Not surprisingly, these diets have been associated with the prevalence of cardiometabolic conditions in the Caribbean^(7)^.

Among ultra-processed food products, sugar-sweetened beverages (SSB) have been identified by international and regional organisations like the WHO, the Healthy Caribbean Coalition and the Caribbean Public Health Association as a major contributor to obesity and cardiometabolic conditions^(8)^. SSB are beverages with sugar added, including soft drinks/soda, flavoured juice drinks, sports drinks, sweetened tea or coffee, energy and electrolyte drinks^(9)^. SSB are extremely energy dense and have almost no nutrient value. Intakes of SSB are highest in the Caribbean (1·9, 95 % CI (1·2, 3·0) servings/d) compared to other regions in the world^(10)^. Data on sales of SSB in the region show a range from 180 to 215 ml/capita weekly^(11)^. Evidence of the negative effect of SSB on health is well established; SSB intake is associated with weight gain, diabetes, hypertension and metabolic syndrome^(12)^.

Though evidence demonstrating the link between SSB and health exists, it is largely limited to populations in high-income countries^(13)^. A World Health Federation Report focused on globalised food systems and health highlighted the paucity of evidence for low- and middle-income countries, including the Caribbean region^(13)^. One main reason has been the absence of systematic regional data collection efforts to generate data that can support ongoing surveillance chronic disease risk factors and outcomes. Establishing an evidence base is critical to regional efforts to reduce SSB intake and associated health conditions. In addition, research on SSB and SSB types is limited in region^(10)^. Further research is essential to support targeted interventions and policies to reduce SSB intake and diet-related disease morbidity.

We sought to address these evidence gaps using data from the Eastern Caribbean Health Outcomes Research Network Cohort Study (ECS). The ECS is a longitudinal cohort established in 2011 to develop a research infrastructure focused on chronic diseases and generate action-oriented research to support policy translation for prevention of chronic diseases^(14)^. Our study objectives were to: (1) describe patterns of SSB consumption by amount and type, (2) examine cross-sectional associations between added sugar from SSB and type 2 diabetes and (3) explore the mediating effect of obesity in the relationship between added sugar from beverages (SSB) and type 2 diabetes.

## Methods

### Data source and sample

We conducted a cross-sectional analysis of ECS baseline data collected during 2013–2018. The ECS is a population-based cohort of 2961 community-dwelling individuals 40 years or older residing in Barbados, Puerto Rico, Trinidad and Tobago or US Virgin Islands. The overarching aim of the ECS is to identify novel risk and protective factors for chronic diseases. Stratified multi-stage random household sampling was used to empanel the ECS in Barbados, Trinidad and Puerto Rico, and random digit sampling was used in the US Virgin Islands of St. Thomas and St. Croix. Eligible participants were English or Spanish-speaking community-dwelling adults 40 years of age and older, who were residents of the island for at least 10 years, and who intended to live on island for the next 5 years. The sampling frame for each site was identified to ensure the representativeness of underlying populations with regard to race, ethnicity, sex and socio-economic status. Smaller sites sampled across the entire island while larger sites sampled from communities that were representative of the larger population. Exclusion criteria included cognitive impairment and residential instability. The response rate for the ECS baseline assessment was 70 %. At baseline, participants completed a self-administered survey including validated measures of lifestyle factors, health outcomes, medical history, dietary intake and demographic characteristics. Participants also underwent clinical examination during which blood pressure and anthropometric measurements, blood and urine samples and medication information were collected. Additional methodological details for the ECS have been previously reported^(14–16)^.

### Sugar-sweetened beverage intake

We assessed SSB intake using the National Cancer Institute Dietary Screener Questionnaire (DSQ)^(17)^. The screener was adapted in an iterative fashion in consultation with local dietitians, nutritionists and research key-informants on each island site to ensure that questions were understood, and that relevant local examples were included for each of the dietary risk factors in each island. Local dietitians and nutritionists were asked to review the original screener, determine local examples and ensure a variety of foods were available in examples. The screener was translated into Spanish in Puerto Rico by native-Spanish speaker and nutrition investigator familiar with Puerto Rican diet (UCR). This was done in consultation with the nutritionist and the PI (CMN) for Puerto Rico and cognitively tested. The instrument was then back-translated into English for English speakers in that island nation. The adapted questionnaire is included in the Appendix.

ECS participants completed the adapted DSQ (available in supplementary materials) which included questions that assessed added sugars from beverages. The following questions were used to measure added sugar from beverages for participants: (1) ‘During the past month, how often did you drink regular soda that contains sugar?’ (2) During the past month, how often did you have coffee or tea that had sugar or honey added to it? (3) During the past month, how often did you drink sweetened fruit drinks, sports, or energy drinks? (Include fruit juices you made at home and added sugar to or bought at a shop.) Participants were asked to select the response option that best reflected their frequency of consumption from never to six or more times daily. Established DSQ scoring algorithms were used to calculate daily teaspoons of added sugar. Briefly, screener item responses were converted to daily servings. Portion sizes were assigned to daily frequencies based on median portion sizes estimated by sex and age from NHANES 24-h recall data. Teaspoon equivalents for added sugars were then estimated by multiplying daily intake frequencies by the corresponding sex- and age-specific median portion size^(17)^. Total added sugar from beverages and from beverage types was operationalised as quartiles and terciles based on the distribution of consumption.

### Main outcomes

Our main outcome was diabetes status. Diabetes status was assessed by self-report and laboratory assessment during the ECS baseline examination. Participants were asked the question, ‘Has a doctor, nurse, or other health professional ever told you that you have diabetes?’ Fasting plasma glucose and HbA1C were determined by laboratory assessment and DCA Vantage Analyzer point of care machines during the baseline clinical exam. We created three categories for diabetes status (no diabetes, unaware pre-diabetes or diabetes, and known pre-diabetes or diabetes) to capture potential differences in added sugar intake according to the stage of disease.

Three categories of diabetes were created for analyses. The American Diabetes Association diagnostic criteria were used to define categories for pre-diabetes and diabetes^(18)^. Known pre-diabetes or diabetes was defined as self-report of pre-diabetes/diabetes or taking blood sugar-lowering medications. Unknown/unaware pre-diabetes or diabetes was defined as having HbA1C value ≥ 5·7 with no self-report of diabetes or report of blood sugar-lowering medication. Participants with no self-report of diabetes, blood sugar-lowering medications and HbA1C ≤ 5·7 were classified as not having diabetes.

From here on, we will refer to known pre-diabetes or diabetes as pre-diabetes/diabetes. Similarly, unknown pre-diabetes or diabetes will be described as unknown pre-diabetes/diabetes.

### Covariates

Covariates included established risk factors for SSB intake and diabetes. Demographic variables included age, sex and educational attainment that were obtained through self-report. Educational attainment was measured by asking participants to report their highest year of school completed. Responses were collapsed into four categories: (1) less than high school, (2) high school, (3) some college and (4) college and beyond. Perceived economic status was measured using the following question, which was adapted from the World Gallup Poll^®^ Questionnaire: ‘Please look at this figure, with steps numbered from 1 at the bottom to 10 at the top. Suppose the top of the ladder represents the richest people of this island and the bottom represents the poorest people of this island. Taking into consideration your current personal situation, what is the number of the step on which you would place yourself?’

Nutrition and lifestyle variables included self-reported physical activity, food insecurity and smoking. Physical activity was measured using the WHO Global Physical Activity Questionnaire and categorised as low *v*. medium/high activity^(19)^. Household food insecurity within the past 90 d was measured using the 9-item sub-scale for adults from the validated Latin American and Caribbean Food Security Scale (ELCSA)^(20)^. Response options were binary (yes/no), and one point was given for each question with a ‘yes’ response. Responses to the first eight questions on the scale were summed for each participant and ranged from 0 to 8. Those who scored 0 were classified as having no food insecurity, 1–6 as having mild/moderate food insecurity and 7–8 as having severe food insecurity. Smoking was measured as current *v*. never and past smokers as done in prior research^(21)^. Clinical covariates included obesity which was measured by BMI calculated from anthropometric measurements taken during the baseline clinical examination. BMI was calculated by dividing participants weight in kilograms by their weight in metres squared.

### Statistical analysis

We performed descriptive analyses to examine distributions and frequencies of study variables. Chi-square tests, *t*-tests and ANOVA were used to assess associations between independent and dependent variables and assess the potential for confounding. Variables found to be significantly (*P* < 0·05) associated with SSB intake and outcomes in the bivariate and ANOVA were included in multivariate analyses. Logistic regression was used to estimate OR and 95 % CI for diabetes. Models were adjusted for age, sex, educational attainment, economic status, physical activity, smoking and food insecurity. We fitted four separate models to examine the association between total added sugar from beverages and diabetes (i.e. known, unknown, none). First, we determined the odds of diabetes according to total added sugar from beverages. Then, we created an overfitted model including BMI to examine the impact of this variable that is on the causal pathway between added sugar and diabetes. Two additional models were fitted to determine the relationship between SSB beverage type (i.e. soda, fruit/energy drink, coffee/tea) and diabetes outcome categories, including an overfitted model with BMI added. All statistical analyses were performed using the Statistical Analysis System statistical software package, version 9.4, SAS Institute Inc. Data for 2388 participants with complete data were included in analyses.

### Role of funding source

The funder of the study had no role in study design, data collection, data analysis, data interpretation or writing of the report. The corresponding author had full access to all the data in the study and had final responsibility for the decision to submit for publication.

## Results

**Fig. 1:**
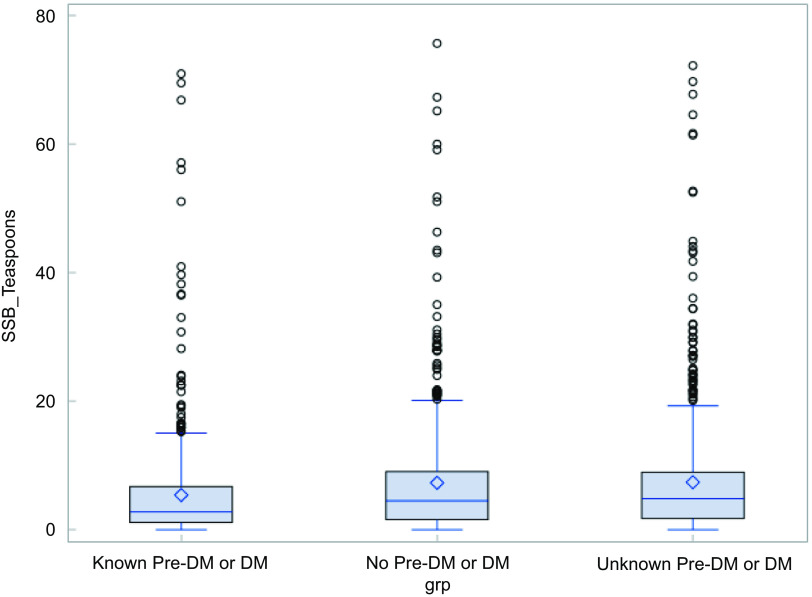
Distribution of added sugar from beverages by diabetes status

The final analytic sample included 1705 who had completed data on main study variables. Sixty-four percentage of participants in the baseline of ECS were women, 32% had less than a high school education and 46% perceived their economic status as ‘average.’ Over 70% of participants were overweight or obese, 46% engaged in low physical activity and 26% experienced food insecurity (see Table 1). Among participants included in the pre-diabetes/diabetes outcome category, 54 % had pre-diabetes. Thirty-six percentage were unaware of their diabetes, 33% were aware of their diabetes and 31% did not have diabetes. The distribution of teaspoons of added sugar by diabetes status is shown in Fig. 1. Results show that overall, 8% of participants reported zero consumption of added sugar from beverages. Participants who were aware of their diabetes were more likely not to consume beverages with added sugar (10 %) compared to those without (7 %) or were unaware (6 %) of their diabetes (*P* = 0·005). Consumption of twelve or more teaspoons was 9 % among those aware, 16 % among those without and 15 % among those with unknown diabetes. Figure 1 shows that median teaspoon consumption of added sugar from beverages was higher among participants who were unaware and who did not have diabetes (five teaspoons) compared to those with diabetes (three teaspoons). Mean values for consumption were higher at eight and seven teaspoons among those without diabetes and those who were unaware, respectively, compared to six teaspoons for those with diabetes.

**Table 1:** Characteristics of ECS cohort participants by diabetes status

Characteristic*	Total (*n* 1705)		No DM or pre-DM (*n* 536)		Known diabetes or pre-diabetes (*n* 558)		Unknown DM or pre-DM (*n* 611)		*P*
	*n*	%	*n*	%	*n*	%	*n*	%	
Socio-demographic characteristics									
	Mean	sd	Mean	sd	Mean	sd	Mean	sd	
Age	57·4 *n*	10·5 %	53·7 *n*	9·8 %	60·6 *n*	10·1 %	57·8 *n*	10·5 %	<0·0001
Age group									<0·0001
40–49	431	25·3	209	39·0	88	15·8	134	21·9	
50–59	591	34·7	191	35·6	165	29·6	235	38·5	
60–69	448	26·3	99	18·5	195	35·0	154	25·2	
70+	235	13·8	37	6·9	110	19·7	88	14·4	
Sex									0·0008
Female	1083	63·5	309	57·7	383	68·6	391	64·0	
Male	622	36·5	227	42·4	175	31·4	220	36·0	
Education									<0·0001
Less than high school	541	31·7	131	24·4	211	37·8	199	32·6	
High school	375	22·0	122	22·8	109	19·5	144	23·6	
Some college	401	23·5	138	25·8	135	24·2	128	21·0	
College+	388	22·8	145	27·1	103	18·5	140	22·9	
Current economic status									0·0227
1–2 (poor)	200	11·7	80	14·9	64	11·5	56	9·2	
3–4	550	32·3	177	33·0	182	32·6	191	31·3	
5–6	775	45·5	216	40·3	254	45·5	305	49·9	
7–8	155	9·1	57	10·6	47	8·4	51	8·4	
9–10 (high)	25	1·5	6	1·1	11	2·0	8	1·3	
Lifestyle and nutritional characteristics									
Current smoker									0·025
No	1554	91·1	474	88·4	518	92·8	562	92·0	
Yes	151	8·9	62	11·6	40	7·2	49	8·0	
BMI									<0·0001
Underweight/normal	440	25·8	183	34·1	119	21·3	138	22·6	
Overweight	621	36·4	197	36·8	182	32·6	242	39·6	
Obese	644	37·8	156	29·1	257	46·1	231	37·8	
Physical activity									0·0002
Low physical activity	801	47·0	225	42·0	297	53·2	279	45·7	
Moderate physical activity	317	18·6	100	18·7	110	19·7	107	17·5	
High physical activity	587	34·4	211	39·4	151	27·1	225	36·8	
Food security status									0·0162
None	1262	74·0	377	70·3	408	73·1	477	78·1	
Low	274	16·1	89	16·6	99	17·7	86	14·1	
Moderate	106	6·2	40	7·5	32	5·7	34	5·6	
Severe	63	3·7	30	5·6	19	3·4	14	2·3	
Location									<0·0001
Barbados	410	24·1	61	11·4	155	27·8	194	31·8	
Puerto Rico	670	39·3	277	51·7	192	34·4	201	32·9	
Trinidad and Tobago	525	30·8	164	30·6	168	30·1	193	31·6	
USVI	100	5·9	34	6·3	43	7·7	23	3·8	

* Chi-square test and ANOVA used for comparisons across groups.

Results (Table 1) showed participants with known pre-diabetes/diabetes status were older compared to those who were unaware of their pre-diabetes/diabetes or did not have pre-diabetes/diabetes (61 years *v*. 58 and 54, respectively) (Table 1). Participants with known diabetes were more likely to be obese (46 %) compared to participants who did not have diabetes or pre-diabetes (29 %) and had unknown diabetes (38 %), respectively. Women and persons with less than a high school education were more likely to have diabetes compared to men and those with greater educational attainment. Moderate food insecurity was highest among persons without pre-diabetes/diabetes compared to those with no diabetes and who were unaware.

**Fig. 2:**
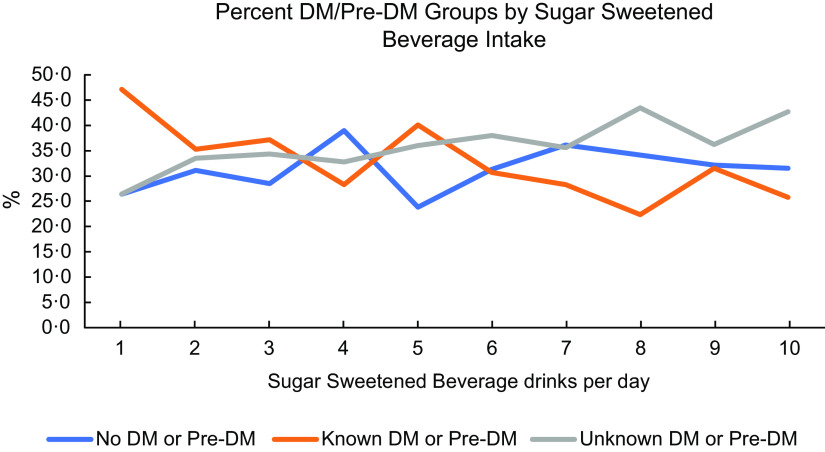
Diabetes status by decile of SSB drinks consumed/d

Mean total intake of added sugar from all beverages and from specific beverage types varied across participant characteristics (Table 2). Total added sugar intake was higher among persons who did not have pre-diabetes/diabetes (mean = 7·6, sd = 11·2) and were unaware (mean = 7·4, sd = 9·8) and compared to those with known pre-diabetes/diabetes (mean = 5·6, sd = 9·1) (*P* = 0·001) Younger (40–49 years old) participants had the highest total intake of added sugar and from each beverage type compared to older persons. Participants who reported current smoking had higher added sugar intake overall compared to those who did not smoke (*P* =< 0·001). Younger participants and men had higher total mean added sugar intake and added sugar from beverage types compared to older persons and women, respectively. Participants who experienced food insecurity had higher total added sugar intake from beverages compared to those who were food secure (*P* < 0·001). Soda and juice drinks were main sources of added sugar among those experiencing food insecurity. Figure 2 illustrates diabetes status according to decile of SSB drinks/d. Results showed that a higher proportion of individuals who were unaware of their diabetes or pre-diabetes were in the highest decile of consumption.

**Table 2:** Socio-demographic and health-related characteristics of ECS participants by mean daily intake of added sugars from SSB

Characteristic	*n*	%	Total (tsp) added sugar from SSB			Daily intake of added sugar (tsp) from soda			Daily intake of added sugar (tsp) from juice and sports			Daily intake of added sugar (tsp) from tea/coffee		
			Mean	sd	*P* *	Mean	sd	*P* *	Mean	sd	*P*	Mean	sd	*P*
Clinical outcome					0·0011			0·1228			0·0016			0·0912
No DM or pre-DM	536	31·4	7·6	11·2		2·7	6·0		2·8	5·8		2·1	2·6	
Known diabetes or pre-diabetes	558	32·7	5·6	9·1		2·1	5·6		1·7	4·6		1·8	2·6	
Unknown DM or pre-DM	611	35·8	7·4	9·8		2·7	5·7		2·6	6·0		2·1	2·7	
Socio-demographic characteristics														
Age group					<0·0001			<0·0001			<0·0001			0·0012
40–49	431	25·3	9·4	13		3·3	6·5		3·8	7·9		2·4	3·2	
50–59	591	34·7	7·4	11·1		3	7·0		2·5	5·6		1·9	2·7	
60–69	448	26·3	4·9	5·9		1·8	3·8		1·5	3·1		1·6	2·1	
70+	235	13·8	4·6	5·4		1·4	3·3		1·1	2·6		2	2·1	
Sex					<0·0001			<0·0001			<0·0001			0·0042
Female	1083	63·5	5·5	8·0		2	5·0		1·7	3·7		1·8	2·4	
Male	622	36·5	9·2	12·7		3·5	6·8		3·6	7·6		2·2	3·0	
Education					0·5485			0·6096			0·0477			0·0132
Less than high school	541	31·7	7	10·6		2·8	6·1		2·6	6·3		1·7	2·5	
High school	375	22·0	7·3	11·6		2·5	5·7		2·8	6·4		2	2·7	
Some college	401	23·5	6·8	8·5		2·5	5·6		2·3	4·4		2·1	2·5	
College+	388	22·8	6·3	9·4		2·2	5·6		1·7	4·5		2·3	2·9	
Current economic status					0·1469			0·3337			0·2513			0·1211
1–2 (poor)	200	11·7	8·4	13·8		3·1	7		3	7·0		2·3	3·0	
3–4	550	32·3	7·1	10·7		2·5	5·6		2·5	6·1		2·0	2·9	
5–6	775	45·5	6·3	8·3		2·3	5·3		2·1	4·8		1·9	2·4	
7–8	155	9·1	6·7	10·5		3·1	6·9		2·1	4·5		1·6	2·0	
9–10 (high)	25	1·5	6·5	9·7		2·5	4·6		1·9	3·8		2·2	2·5	
Lifestyle and nutritional characteristics														
Current smoker					<0·0001			0·409			0·6832			0·1911
No	1554	91·1	6·5	9·8		2·3	5·5		2·2	5·5		1·9	2·6	
Yes	151	8·9	10·6	12·7		4·2	7·9		3·5	6·2		2·8	3·3	
BMI					0·346			0·0001			0·0073			<0·0001
Underweight/normal	440	25·8	6·3	9·9		2·3	5·9		2·2	4·7		1·8	2·3	
Overweight	621	36·4	6·9	10·0		2·4	5·5		2·4	5·3		2·1	2·8	
Obese	644	37·8	7·2	10·4		2·8	5·9		2·5	6·3		2·0	2·7	
Physical activity					0·002			0·0929			0·0003			0·1221
Low physical activity	801	47·0	6·4	8·4		2·5	5·5		1·9	4·4		2·0	2·6	
Moderate physical activity	317	18·6	5·7	8·1		1·9	4·5		2·1	4·7		1·7	2·2	
High physical activity	587	34·4	8·0	12·8		2·8	6·7		3·1	7·1		2·1	2·9	
Food security status					<0·0001			<0·0001			<0·0001			0·0674
None	1262	74·0	6·0	8·1		2·1	4·9		1·9	4·7		1·9	2·4	
Low	274	16·1	9·2	14·8		3·8	8·1		3·3	7·0		2·1	3·1	
Moderate	106	6·2	10·2	13·5		3·2	7·0		4·4	8·7		2·6	3·5	
Severe	63	3·7	8·9	12·2		4·0	7·0		2·9	4·9		2·0	2·8	

* Chi-square test and ANOVA used for comparisons across groups.

Table 3 presents logistic regression model results for diabetes outcomes. Models 1–4 were adjusted for age, sex, educational attainment, economic status, physical activity and food insecurity. Models 2 and 4 also included BMI. Model 1 results showed that total added sugar from beverages was not associated with diabetes status. The inclusion of obesity (model 2) did not change the direction of associations or result in statistically significant findings.

**Table 3:** Logistic regression model results for the relationship between added sugar from beverages and diabetes

Models	DM or pre-DM (known or unknown) *v*. no DM or pre-DM				Known DM or pre-DM *v*. no DM or pre-DM				Unknown DM or pre-DM *v*. no DM or pre-DM			
	OR	95 % CI		*P*	OR	95 % CI		*P*	OR	95 % CI		*P*
Model 1												
SSB – Quartile 2 (1·55–4·12 TSP) *v*. Quartile 1 (0–1·54 TSP)	0·88	0·65	1·19	0·4072	0·89	0·63	1·27	0·5141	0·90	0·64	1·29	0·5736
SSB – Quartile 3 (4·13–8·50 TSP) *v*. Quartile 1 (0–1·54 TSP)	1·05	0·77	1·43	0·7791	0·85	0·58	1·22	0·3739	1·27	0·89	1·81	0·1826
SSB – Quartile 4 (> 8·50 TSP) *v*. Quartile 1 (0–1·54 TSP)	1·04	0·75	1·43	0·8225	0·78	0·53	1·16	0·2166	1·31	0·91	1·88	0·1440
Model 2*												
SSB – Quartile 2 (1·55–4·12 TSP) *v*. Quartile 1 (0–1·54 TSP)	0·85	0·62	1·16	0·3020	0·85	0·59	1·22	0·3808	0·88	0·62	1·26	0·4824
SSB – Quartile 3 (4·13–8·50 TSP) *v*. Quartile 1 (0–1·54 TSP)	1·01	0·73	1·39	0·9576	0·78	0·53	1·13	0·1881	1·25	0·88	1·79	0·2162
SSB – Quartile 4 (> 8·50 TSP) *v*. Quartile 1 (0–1·54 TSP)	0·95	0·69	1·32	0·7611	0·72	0·49	1·07	0·1051	1·22	0·85	1·76	0·2799
Model 3												
Sugar-sweetened soda – Quartile 2 (>0–0·66 TSP) *v*. Quartile 1 (0 TSP)	1·26	0·92	1·71	0·1461	1·12	0·78	1·61	0·5328	1·36	0·96	1·92	0·0852
Sugar-sweetened soda – Quartile 3 (0·67–2·57 TSP) *v*. Quartile 1 (0 TSP)	1·39	1·06	1·81	0·0179	1·29	0·93	1·79	0·1213	1·48	1·09	2·00	0·0123
Sugar-sweetened soda – Quartile 4 (> 2·57 TSP) *v*. Quartile 1 (0 TSP)	1·37	1·03	1·82	0·0331	1·18	0·83	1·68	0·3613	1·54	1·12	2·13	0·0080
Sugar-sweetened fruit drink, energy drink – middle third (>0–1·78 TSP) *v*. bottom third (0 TSP)	1·21	0·93	1·57	0·1485	1·05	0·77	1·43	0·7680	1·38	1·03	1·85	0·0309
Sugar-sweetened fruit drink, energy drink – Top third (> 1·78 TSP) *v*. bottom third (0 TSP)	0·97	0·76	1·25	0·8189	0·72	0·53	0·97	0·0315	1·22	0·92	1·61	0·1699
Sugar in coffee – Quartile 2 (>0–1·41 TSP) *v*. Quartile 1 (0 TSP)	1·01	0·76	1·34	0·9684	0·86	0·61	1·20	0·3733	1·18	0·86	1·63	0·3136
Sugar in coffee – Quartile 3 (1·42–2·15 TSP) *v*. Quartile 1 (0 TSP)	0·77	0·58	1·02	0·0654	0·64	0·46	0·89	0·0077	0·92	0·67	1·28	0·6297
Sugar in coffee – Quartile 4 (> 2·15 TSP) *v*. Quartile 1 (0 TSP)	0·80	0·60	1·06	0·1144	0·65	0·46	0·91	0·0130	0·93	0·68	1·28	0·6475
Model 4*												
Sugar-sweetened soda – Quartile 2 (>0–0·66 TSP) *v*. Quartile 1 (0 TSP)	1·17	0·86	1·60	0·3234	1·04	0·72	1·51	0·8268	1·29	0·90	1·83	0·1609
Sugar-sweetened soda – Quartile 3 (0·67–2·57 TSP) *v*. Quartile 1 (0 TSP)	1·29	0·98	1·70	0·0737	1·20	0·86	1·68	0·2862	1·36	1·00	1·85	0·0520
Sugar-sweetened soda – Quartile 4 (> 2·57 TSP) *v*. Quartile 1 (0 TSP)	1·23	0·92	1·65	0·1715	1·05	0·73	1·52	0·7779	1·42	1·03	1·97	0·0340
Sugar-sweetened fruit drink, energy drink – middle third (>0–1·78 TSP) *v*. bottom third (0 TSP)	1·24	0·95	1·61	0·1167	1·04	0·76	1·43	0·8003	1·44	1·07	1·94	0·0160
Sugar-sweetened fruit drink, energy drink – top third (> 1·78 TSP) *v*. bottom third (0 TSP)	0·98	0·76	1·27	0·9003	0·72	0·53	0·98	0·0356	1·23	0·92	1·63	0·1630
Sugar in coffee – Quartile 2 (>0–1·41 TSP) *v*. Quartile 1 (0 TSP)	0·97	0·72	1·29	0·8252	0·79	0·56	1·12	0·1926	1·15	0·83	1·60	0·3949
Sugar in coffee – Quartile 3 (1·42–2·15 TSP) *v*. Quartile 1 (0 TSP)	0·73	0·55	0·97	0·0298	0·58	0·41	0·81	0·0014	0·89	0·64	1·24	0·4999
Sugar in coffee – Quartile 4 (> 2·15 TSP) *v*. Quartile 1 (0 TSP)	0·78	0·58	1·03	0·0838	0·61	0·43	0·87	0·0065	0·92	0·67	1·28	0·6271

* Models 2 and 4 also adjusted for BMI.

All models adjusted for age, sex, education, current economic status, smoking status, physical activity, and food security.

Models 3 results according to beverage type (excluding BMI) showed statistically significant relationships across diabetes outcomes. Participants in the highest consumption quartiles of sugar from soda had 39 and 37% greater odds of having diabetes compared to those in the lowest consumption quartile (*P* < 0·05). Results were similar for unknown diabetes and showed that participants in the third and fourth quartiles of added sugar from soda (*v*. quartile 1) had greater odds of unknown pre-diabetes/diabetes compared to participants without diabetes (OR = 1·48, 95 % CI (1·09, 2·00); OR = 1·54, 95 % CI (1·12, 2·13)). Added sugar from energy drinks/fruit drinks was inversely associated with known diabetes among those in the highest consumption tercile (*v*. tercile 1) (OR = 0·72, 95 % CI (0·53, 0·97)). In contrast, results for unknown diabetes showed that participants in the middle tercile of added sugar (*v*. tercile 1) from juice/energy drinks had greater odds of unknown pre-diabetes/diabetes compared to individuals without diabetes (OR = 1·38, 95 % CI (1·03, 1·85)). Added sugar from coffee/tea was only associated with the outcome known diabetes. Results showed that persons in quartiles 3 and 4 had 36 and 35% lower odds of known diabetes, respectively. The addition of BMI in model 4 showed similar associations remained statistically significant though OR were attenuated.

## Discussion

In this cross-sectional study, we aimed to assess consumption of added sugar from SSB and examine associations with type II diabetes. We also examined the potential mediating role of obesity and role of food insecurity in these associations. Our results showed that soda was a main source of added sugar from beverages, intake varied across socio-demographic characteristics and consumption of added sugar from specific beverage types was independently associated with diabetes. Findings also showed this association was not meaningfully influenced by obesity or food insecurity. Other notable findings included the increased consumption of added sugar from beverages among participants who experienced food insecurity and the positive association between food insecurity and known diabetes.

Beverage patterns observed in our study were consistent with prior studies of SSB intake, globally and in region, that demonstrated higher than recommended overall intakes, higher intakes among younger people^(22)^ and soda as a major SSB beverage source^(23)^. Findings were also consistent with prior literature that showed an independent association between added sugar beverage consumption and diabetes^(24)^; however, the current study departs from prior findings in the direction of the association. Most existing studies of added sugar from beverages and diabetes are prospective and consistently demonstrated a positive association between SSB consumption and diabetes, even after adjustment for energy intake and BMI^(13,24,25)^. The cross-sectional nature of our study and contrary finding of an inverse association between added sugar from beverages (SSB) and known diabetes suggests reverse causality. This is likely a result of dietary changes that occurred in response to being diagnosed with diabetes. Nutritional counselling is an important component of post-diagnosis counselling for diabetes, and evidence shows that people make dietary changes following a diagnosis^(26)^. Our cross-sectional design precludes the ability to assess temporality in the relationship between added sugar and diabetes status.

A notable finding was that 36% of participants with elevated HA1C suggestive of pre-diabetes or diabetes were unaware of their status and had total mean intakes of added sugar (mean 7·4, sd = 9·8) that were close to or exceeded maximum recommended levels. Mean added sugar intakes from beverages alone were above the American Heart Association recommended daily limit of nine and six teaspoons for men and women, respectively^(27)^. Our estimates of added sugar from beverages alone also suggest that overall added sugar intake in participant diets is higher than the maximum recommended by the WHO of no more than 10 % of a 2000 calorie diet (12 tsp). One study conducted in region showed that only 22 and 26% of men and women met the USDA and WHO recommendation of less than 10% of energy from added sugar^(22)^. Compared to other food sources, SSB contributed the highest percentage to total energy intake. Though findings are from a single island, we believe the impact of SSB is similar on other islands given their similar stage of epidemiologic transition. Higher consumption of added sugar and greater odds of diabetes among persons who experience food insecurity is also notable. This is likely a result of changes to food systems in the region that influence increased availability of foods containing added sugars which we know have health implications. Our findings suggest a critical need for primary diabetes prevention, screening and attention to social needs like food insecurity.

The heavy reliance on imported food in the Caribbean region threatens food security, defined as access to safe, sufficient and nutritious foods that meet food preferences and dietary needs^(28)^. High costs of imported food have led to decreased affordability of healthy foods especially among individuals experiencing food security^(29)^ and are implicated in dietary intakes that are consequential for chronic disease. Changes in the food system and parallel increases in cardiometabolic conditions, like diabetes, are implicated in premature mortality, decreased economic productivity and decreased quality of life in the region^(7)^. This cascade effect of unhealthy dietary changes, including increased SSB consumption, prompted the creation of initiatives and policies to increase healthy food environments and improve diets^(30)^.

Taken together, current evidence and international recommendations strongly support the critical importance of reducing SSB intake in the Caribbean region. Regional efforts to reduce SSB intake have included banning SSB sales in schools and instituting taxation of SSB^(2)^. The latter is just one of several WHO endorsed fiscal policy solutions aimed to reduce obesity and other metabolic diseases including diabetes^(31)^. Assessment of the impact of the SSB tax on beverage sales showed that SSB tax was effective at reducing sales of SSB by 4·3% after the first year of implementation^(32)^. However, the health impact of SSB consumption has not yet been established in the region.

Though regional public health organisations and governments have prioritised SSB reduction, there is an urgent need for a more widespread and robust response to achieve population-wide shifts in SSB intake. Action is needed to address key contributing factors such as the influx of imported foods high in sugar, salt and fat and the lack of incentives for consumption of healthier foods. There is also a need for the promotion of alternatives to SSB consumption, especially water, to move beyond education on SSB for individuals and communities that are most often already knowledgeable about the perils of unhealthy dietary intakes. Policies to support increased screening for diabetes are important to lessen CVD burden associated with high prevalence of diabetes in the region. Future research is needed to examine the longitudinal relationship between SSB and diabetes in the Caribbean and identify effective food environment interventions that promote healthier food choices.

Our study has potential limitations. The use of DSQ algorithms, which are based on consumption patterns in the USA, has not been validated in Caribbean populations and therefore may not accurately reflect SSB intake frequency in the Caribbean. Based on the results of global and regional studies of SSB consumption, SSB consumption in the Caribbean is similar or greater than consumption in the USA^(10)^. Therefore, it is possible that our results are an underestimate of true SSB consumption in the region. The ECS cohort includes participants 40 years and older which precluded the ability to examine consumption among younger persons who are high consumers of SSB^(10,22)^. Despite these limitations, this study provides needed contextually relevant evidence on SSB intake in a multisite Caribbean cohort. This evidence is essential to support existing and future initiatives to reduce SSB intake in the region.

## Supplementary material

For supplementary material accompanying this paper visit https://doi.org/10.1017/S1368980023000381. click here to view supplementary material
