# Supplementary material for: Consumption of sugar-sweetened beverages and T2D diabetes in the Eastern Caribbean
Source: Public Health Nutr. 2023 Mar 1;26(7):1403–13. doi: 10.1017/S1368980023000381 (PMC10346089; doi:10.1017/S1368980023000381)
Supplement: Supplementary file 1 [file S1368980023000381sup001.docx]

**Appendix**

Questions sourced from the National Cancer Institute Dietary Screener Questionnaire

During the past month, how often did you drink regular soda that contains Sugar (such as Ting or ginger beer, or others)? Do not include diet soda.

[ ] Never

[ ] 1 time last month

[ ] 2 to 3 times last month

[ ] 1 time per week

[ ] 2 times per week

[ ] 3 to 4 times per week

[ ] 5 to 6 times per week

[ ] 1 time per day

[ ] 2 or more times per day

[ ] 4 to 5 times per day

[ ] 6 or more times per day

During the past month, how often did you drink 100% pure fruit juice without added sugar, such as apple, orange, grape, pineapple and mango juices?

[ ] Never

[ ] 1 time last month

[ ] 2 to 3 times last month

[ ] 1 time per week

[ ] 2 times per week

[ ] 3 to 4 times per week

[ ] 5 to 6 times per week

[ ] 1 time per day

[ ] 2 or more times per day

[ ] 4 to 5 times per day

[ ] 6 or more times per day

During the past month, how often did you have coffee or tea that had sugar or honey added to it? Include coffee and tea you sweetened yourself, bought at a coffee shop and presweetened tea and coffee drinks.

[ ] Never

[ ] 1 time last month

[ ] 2 to 3 times last month

[ ] 1 time per week

[ ] 2 times per week

[ ] 3 to 4 times per week

[ ] 5 to 6 times per week

[ ] 1 time per day

[ ] 2 or more times per day

[ ] 4 to 5 times per day

[ ] 6 or more times per day

During the past month, how often did you drink sweetened fruit drinks, sports or energy drinks? Include fruit juices you made at home and added sugar to, or bought at a shop, such as tamarind, lemonades or passion fruit juices.

[ ] Never

[ ] 1 time last month

[ ] 2 to 3 times last month

[ ] 1 time per week

[ ] 2 times per week

[ ] 3 to 4 times per week

[ ] 5 to 6 times per week

[ ] 1 time per day

[ ] 2 or more times per day

[ ] 4 to 5 times per day

[ ] 6 or more times per day
